# Demographic Patterns and Outcomes of Patients in Level I Trauma Centers in Three International Trauma Systems

**DOI:** 10.1007/s00268-015-3162-x

**Published:** 2015-07-17

**Authors:** Amy C. Gunning, Koen W. W. Lansink, Karlijn J. P. van Wessem, Zsolt J. Balogh, Frederick P. Rivara, Ronald V. Maier, Luke P. H. Leenen

**Affiliations:** Department of Trauma Surgery, University Medical Center Utrecht, Suite: G04.228, Heidelberglaan 100, 3584 CX Utrecht, The Netherlands; Department of Traumatology, John Hunter Hospital and University of Newcastle, Newcastle, Australia; Department of Pediatrics, Epidemiology, and Harborview Injury Prevention and Research Center, University of Washington, Seattle, WA USA; Department of Trauma, Burns and Critical Care Surgery, Harborview Medical Center, University of Washington, Seattle, WA USA

## Abstract

**Introduction:**

Trauma systems were developed to improve the care for the injured. The designation and elements comprising these systems vary across countries. In this study, we have compared the demographic patterns and patient outcomes of Level I trauma centers in three international trauma systems.

**Methods:**

International multicenter prospective trauma registry-based study, performed in the University Medical Center Utrecht (UMCU), Utrecht, the Netherlands, John Hunter Hospital (JHH), Newcastle, Australia, and Harborview Medical Center (HMC), Seattle, the United States. Inclusion: patients ≥18 years, admitted in 2012, registered in the institutional trauma registry.

**Results:**

In UMCU, JHH, and HMC, respectively, 955, 1146, and 4049 patients met the inclusion criteria of which 300, 412, and 1375 patients with Injury Severity Score (ISS) > 15. Mean ISS was higher in JHH (13.5; *p* < 0.001) and HMC (13.4; *p* < 0.001) compared to UMCU (11.7). Unadjusted mortality: UMCU = 6.5 %, JHH = 3.6 %, and HMC = 4.8 %. Adjusted odds of death: JHH = 0.498 [95 % confidence interval (CI) 0.303–0.818] and HMC = 0.473 (95 % CI 0.325–0.690) compared to UMCU. HMC compared to JHH was 1.002 (95 % CI 0.664–1.514). Odds of death patients ISS > 15: JHH = 0.507 (95 % CI 0.300–0.857) and HMC = 0.451 (95 % CI 0.297–0.683) compared to UMCU. HMC = 0.931 (95 % CI 0.608–1.425) compared to JHH. TRISS analysis: UMCU: Ws = 0.787, Z = 1.31, *M* = 0.87; JHH, Ws = 3.583, *Z* = 6.7, *M* = 0.89; HMC, Ws = 3.902, *Z* = 14.6, *M* = 0.84.

**Conclusion:**

This study demonstrated substantial differences across centers in patient characteristics and mortality, mainly of neurological cause. Future research must investigate whether the outcome differences remain with nonfatal and long-term outcomes. Furthermore, we must focus on the development of a more valid method to compare systems.

## Introduction

Trauma systems were developed during the last 40 years to coordinate and improve the care for the injured [1]. A regionalized care approach was established with a combination of levels of designated trauma centers. Evaluations have demonstrated the efficacy in terms of better triage and improved patient outcomes [2–5].

The verified trauma centers in a trauma system follow the criteria outlined by the American College of Surgeons Committee on Trauma (ACS-COT) [1]. Although the aim of a trauma system is similar in each country, major differences and variations exist in the designation and elements comprising the system both within and across countries. For instance, differences in geographical service areas, trauma mechanisms, demographic injury patterns, trauma patient volumes, and trauma resources, such as the availability of dedicated trauma teams, trauma surgeons, and operation facilities. Each of these factors may have an influence on patient characteristics and the outcome of patients. Lessons can be learned from different system designs, therefore it is important for trauma systems to compare and benchmark other systems.

In this study, we examine three international trauma systems by comparing the demographic patterns and patient outcomes in three Level I trauma centers.

## Methods and patient setting

### Study design

We performed an international multicenter trauma registry-based study with prospectively collected data at three Level I trauma centers functioning within verified trauma systems:

- University Medical Center Utrecht (UMCU), Utrecht, the Netherlands.
- John Hunter Hospital (JHH), Newcastle, Australia.
- Harborview Medical Center (HMC), Seattle, United States.

Each tertiary care facility has a central role and leadership within a trauma system and has adequate depth of resources and personnel to care for the most severely injured patients [1]. Data on all trauma admissions are registered in the institutional trauma registry and the national trauma registry, which includes the same variables as the Major Trauma Outcome Study database (MTOS) [6].

This study is conducted in accordance with the principles of the Declaration of Helsinki [7] and Good Clinical Practice Guidelines [8]. The Institutional Review Board of the UMCU, JHH, and HMC approved the study.

#### University Medical Center Utrecht

In 1999, regionalized trauma care was instituted in the Netherlands. In the Dutch trauma system, 11 Level I trauma centers were established, each covering a specific region in the Netherlands. The UMCU officially became a Level I trauma center in 2000 and covers the central region of the Netherlands. Four Level II and III trauma centers are connected to this network. The longest distance between the centers is approximately 50 km. The Medical Air Assistance of the Royal Dutch Touring Club (ANWB) provides the prehospital care in the air, and the Regional Ambulance Care Utrecht (RAVU) on the road.

The trauma registry includes all direct trauma admissions from the emergency department (ED).

#### John Hunter Hospital

The first introduction of a system for trauma care in Australia was in New South Wales (NSW); in 1988, it became Australia’s first state trauma plan and was implemented in NSW in 1992 [9]. JHH is a state-designated Level I trauma center, verified by the Royal Australasian College of Surgeons. It is the only major tertiary referral hospital for the Hunter New England region. The John Hunter trauma service was established in 2005.

The prehospital care in the Hunter region is provided by the Ambulance Service of NSW and utilizes both road and helicopter primary retrieval from the trauma scene. Two helicopters serve the area. By protocol, all major trauma patients in the Hunter New England region are transported to the Level I trauma center.

All trauma patients registered in the trauma registry had a Full Trauma Team Activation or an ISS > 15.

#### Harborview Medical Center

The first trauma systems were developed in the United States (US) in the late 1960s [10, 11]. HMC was the first designated Level I trauma center in the state of Washington and verified in 1993. It serves as the only Level I trauma center in four states, Washington, Alaska, Idaho, and Montana. The majority of the trauma patients, approximately 95 %, come from the state of Washington.

The prehospital transportation is provided by ground ambulances from the Seattle Emergency Medical Service (EMS) system and the King County EMS system, and the air ambulance system is managed by Airlift Northwest [12].

In HMC, all trauma admissions are registered in the institutional trauma registry, except for patients aged ≥65 years with an isolated neck of femur fracture.

An overview of the key differences of the three trauma centers is presented in Table 1 [13–19].

**Table 1:** Key demographic and trauma center differences

	UMCU	JHH	HMC
Country	The Netherlands	Australia	The United States
Service area (km^2^)	1500	130,000	185,000
Residents (n)	1,300,000	840,000	7,000,000
Verification level trauma center	Level I	Level I	Level I
Annually total hospital patient volume	±35000	±40000	±19000
Annually trauma patient volume	±1300	±4500	±6000
Annually trauma patient volume, ISS > 15	±375	±425	±2000
Surgeons involved in acute trauma care	6	4	10

*km* kilometers, *n* number of patients, and *ISS* Injury Severity Score

### Patients

All consecutive patients, aged 18 years and older, with blunt or penetrating injury admitted to each of the trauma centers between January 1, 2012 and December 31, 2012 were selected from the institutional trauma registry. Patients dead on arrival in ED, or with injuries due to burns, electrocution, or drowning were excluded.

### Data

Data were collected from the institutional trauma registry and electronic medical records. The collected data were age, gender, trauma mechanism, Glasgow Coma Score (GCS), systolic blood pressure (SBP), respiratory rate (RR), Revised Trauma Score (RTS), Abbreviated Injury Scale (AIS) score (version 2005), Injury Severity Score (ISS), survival probability, hospital length of stay (H-LOS) in days, intensive care unit length of stay (ICU-LOS) in days, in-hospital mortality, and cause of death.

A patient with an ISS above 15 was considered a severely injured patient. Patients with an AIS score in the head region were noted as a patient with neurotrauma, an AIS > 3 was scored as severe neurotrauma.

### Statistical analysis

We used multiple imputation methods for missing data of the physiological parameters (i.e., GCS, SBP, and RR). Studies have demonstrated that multiple imputation leads to less biased results [20]. Furthermore, we have demonstrated in a previous study that multiple imputation is a reliable method despite the percentages of missing data [21]. In UMCU, GCS, SBP, and RR were missing in, respectively, 29.2, 21.9, and 65.3 % of the patients. In JHH, GCS, SBP, and RR were missing in, respectively, 14.8, 11.3, and 11.4 % of the patients. In HMC, in 12.0, 0.2, and 1.1 % of the patients, respectively, GCS, SBP, and RR were missing.

Trauma and Injury Severity Score (TRISS) methodology was used to compare the trauma center performances. We have calculated the standardized Ws score, proposed by Hollis et al. [22]., Z score, and M statistic. The Ws score states the number of excess survivors compared to the baseline database (MTOS database) per 100 patients [6]. The significance of the Ws score is determined by the Z score, a value below −1.96 and above 1.96 indicates, respectively, a significantly worse and better performance. The M statistic describes the injury severity mix between the studied institution and the baseline database, a value below 0.88 indicates a disparity in the severity match between the two groups [23].

A multivariable logistic regression model adjusted for confounders was used to calculate odds ratio (OR). The OR was used as an estimate of the relative risk of death, given the outcome was rare. The covariates adjusted for were age, ISS, RTS, and severe neurotrauma, all parameters known to influence the outcome substantially. To achieve comparable populations, we have standardized the inclusion criteria and performed a subanalysis for the odds of deaths in patients with ISS > 15.

Continuous variables were compared with independent sample Student’s t-test and the Mann–Whitney U-test. Categorical variables were compared with the Chi-square test. Mean values are presented with their standard deviations (SD) and medians with their interquartile range (IQR).

The imputation of missing data and the statistical analysis were performed with SPSS, version 20.0 (IBM Corp., Armonk, NY) for Windows. Significance of statistical differences was attributed to p < 0.05.

## Results

**Fig. 1:**
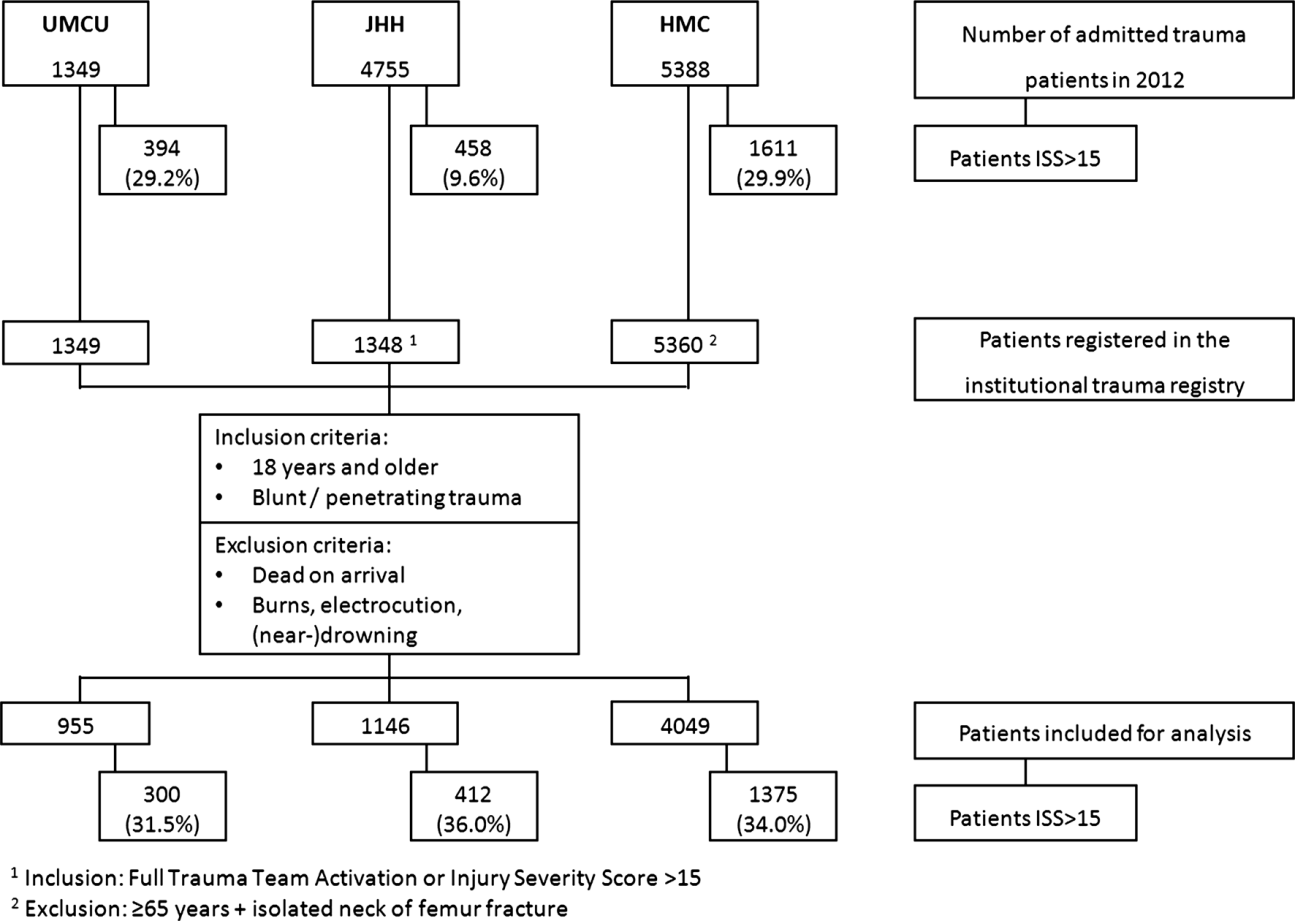
Flowchart of patients included for analysis. ^1^Inclusion: Full Trauma Activation or injury severity score >15. ^2^Exclusion: ≥65 years + isolated neck of femur fracture

An overview of the study patients is shown in Fig. 1. In total, 955 patients met the inclusion criteria from UMCU, 1146 patients from JHH, and 4049 patients from HMC. Patients in UMCU were slightly older, more likely to be female and had longer hospital lengths of stay. Penetrating trauma was more common at HMC compared to UMCU (*p* < 0.001) and JHH (*p* < 0.001). Compared to the UMCU population mean, ISS was higher in both JHH (*p* < 0.001) and HMC (*p* < 0.001). The proportion of patients with neurotrauma was highest in JHH followed by UMCU and HMC. UMCU had the highest proportion of patients with severe neurotrauma. Almost 50 % of the patients in HMC were admitted to the ICU in contrast to 20.6 % in UMCU and 15.8 % in JHH. Though the ICU patients in UMCU and JHH were more severely injured [median ISS, respectively, 21 (13–27) and 25 (17–34)] compared to HMC [median ISS 17 (10–26)]. Unadjusted mortality was significantly higher at UMCU compared to JHH and HMC. All these patient characteristics are presented in Table 2.

**Table 2:** Patient characteristics

	UMCU	JHH	HMC
Patients, n	955	1146	4049
Patients ISS > 15 (n) (%)	300 (31.5)	412 (36.0)^a^	1375 (34.0)
Type of injury			
Blunt (n) (%)	889 (93.1)	1086 (94.8)	3508 (86.6)^ab^
Penetrating (n) (%)	66 (6.9)	60 (5.2)	541 (13.4)^ab^
Mean age (SD)	51.5 (21.057)	45.7 (20.284)^a^	49.5 (20.599)^ab^
Gender			
Male (n) (%)	608 (63.6)	836 (72.9)^a^	2741 (67.7)^ab^
Female (n) (%)	348 (36.4)	310 (27.1)^a^	1308 (32.2)^ab^
ISS			
Mean (SD)	11.7 (9.735)	13.5 (10.514)^a^	13.4 (10.977)^a^
Median (IQR)	9 (4–17)	10 (5–17)^a^	10 (5–17)^a^
ISS, patients ISS > 15			
Mean (SD)	23.4 (7.551)	24.4 (9.940)^a^	25.5 (10.170)^a^
Median (IQR)	21 (17–26)	22 (17–28.5)^a^	22 (17–29)^a^
ISS subgroups			
ISS < 9 (median, IQR)	4 (2–5)	5 (4–6)^a^	5 (4–5)^a^
ISS 9–15 (median, IQR)	10 (9–12)	10 (9–13)^a^	10 (9–12)^a^
ISS 16–24 (median, IQR)	17 (17–21)	17 (17–21)	17 (17–21)
ISS 24–40 (median, IQR)	27 (25–29.75)	29 (25–33)	29 (26–33)^a^
ISS > 40 (median, IQR)	43 (41–49)	50 (45–57)^a^	48 (43–57)^a^
RTS, mean (SD)	7.31 (0.997)	7.44 (0.964)^a^	7.17 (1.316)^a^
Mean Ps (SD)	0.92 (0.162)	0.93 (0.154)^a^	0.90 (0.194)^ab^
Neurotrauma (n) (%)	457 (47.9)	690 (60.2)	1702 (42.0)
Mild (AIS ≤ 3) (n) (%)	261 (27.3)	481 (42.0)^a^	989 (24.4)^b^
Severe (AIS ≥ 4) (n) (%)	196 (20.5)	209 (18.2)	712 (17.6)^a^
ICU admission (n) (%)	197 (20.6)	181 (15.8)^a^	1954 (48.3)^ab^
H-LOS, days			
Mean (SD)	10.5 (13.331)	8.9 (14.347)^a^	8.2 (11.301)^a^
Median (IQR)	6 (2–13)	5 (3–9)^a^	5 (2–9)^a^
ICU-LOS, days			
Mean (SD)	7.0 (10.438)	6.7 (6.995)	5.4 (5.853)
Median (IQR)	3 (2–9)	4 (2–8)	3 (3–6)
Mortality	62 (6.5)	41 (3.6)^a^	194 (4.8)^a^
Mortality, patients ISS > 15	54 (18 %)	40 (9.7 %)^a^	171 (12.4 %)^a^

^a^significantly different from UMCU

^b^significantly different from JHH

After adjustment, the OR for mortality for all patients at JHH and HMC was 0.498 [95 % confidence interval (CI) 0.303–0.818] and 0.473 (95 % CI 0.325–0.690), respectively, compared to UMCU. The odds for death in HMC compared to JHH was 1.002 (95 % CI 0.664–1.514).

For the severely injured patients, the adjusted ORs for death were 0.507 (95 % CI 0.300–0.857) in JHH and 0.451 (95 % CI 0.297–0.683) in HMC compared to UMCU (Table 3). The odds for death for the severely injured patients in HMC was 0.931 (95 % CI 0.608–1.425) compared to JHH. Unadjusted causes of death are presented in Table 4.

**Table 3:** Adjusted odds for death, OR (95 % CI)

	All patients	Patients ISS > 15
UMCU versus JHH	0.498 (0.303–0.8180), *p* = 0.006	0.507 (0.300–0.857), *p* = 0.011
UMCU versus HMC	0.473 (0.325–0.690), *p* < 0.001	0.451 (0.297–0.683), *p* < 0.001
JHH versus HMC	1.002 (0.664–1.514), *p* = 0.991	0.931 (0.608–1.425), *p* = 0.742

**Table 4:** Causes of death

	UMCU	JHH	HMC
Exsanguination	3 (4.8)	4 (9.8)	14 (7.2)
CNS injury	42 (67.7)	33 (80.5)^a^	115 (59.3)^a^
Resp failure/PNA/ARDS	8 (12.9)	1 (2.4)^a^	37 (19.1)^b^
Sepsis	0	0	4 (2.1)
SOF/MOF	5 (8.1)	1 (2.4)	7 (3.6)^a^
Cardiac	0	2 (4.9)	8 (4.1)
Multiple injuries	2 (3.2)	0	5 (2.6)
Other (CVA)	0	0	1 (0.5)
Unknown	2 (3.2)	0	3 (1.5)

^a^significantly different from UMCU

^b^significantly different from JHH

In Table 5, we observed a positive number of excess survivors in JHH and HMC compared to the baseline population. TRISS analysis in the UMCU population showed no significant difference from the baseline database, and therefore the number of excess survivors equals zero. The M score of UMCU and HMC was below the allowed value of 0.88.

**Table 5:** TRISS analysis

	UMCU	JHH	HMC
Ws	0.787	3.583	3.902
*Z*	1.31	6.7	14.6
*M*	0.87	0.89	0.84

## Discussion

In this study, we have described differences between international trauma systems by comparing the demographic patterns and outcomes of trauma patients in three international Level I trauma centers.

A significant difference in the results was the survival rates at the trauma centers. The crude mortality in UMCU was significantly higher compared to JHH and HMC. Adjusted for confounders, the odds for death in JHH and HMC was much lower compared to UMCU (Table 3). Both crude and adjusted mortality did not significantly differ between JHH and HMC.

The difference was also demonstrated with the TRISS method. These analyses showed that both HMC and JHH performed better than the international standard. The performance of UMCU was equal to this standard. (Table 5) Based on these results, we could state that HMC and JHH performed better than UMCU. However, at both HMC and UMCU, the M score was just below its threshold value, suggesting a disparity in injury severity match in these populations. Several authors have stated that TRISS has become an inappropriate tool to compare trauma center performances. It has an unacceptably high misclassification rate in severely injured patients, and the TRISS coefficients are drifting out of calibration [24–26]. Furthermore, TRISS is thought to underestimate the severity in penetrating injury and could have caused an underestimation of the W and Z score. TRISS is also known to overestimate the severity in patients who are intubated for presumed head injury in the presence of intoxicating substance. This could not be addressed in this study because we did not have these specific patient data.

Furthermore, the large differences between service areas in the various countries might influence the prehospital times substantially. The prehospital times in JHH and HMC are longer, therefore similarly injured patients might have worse physiological parameters at the time of arrival which influences the RTS [23]. Because of the substantial weight of the RTS in the TRISS model, this might lead to different mortality predictions in similar patients and qualify a trauma center incorrectly as an outlier [27].

Though the TRISS methodology is limited, it is the only benchmark we currently have across separate trauma systems. We have to readdress and update the TRISS method and search for a solution to cope with the demographic differences in the trauma populations.

The differences in mortality in this study could be partially assigned to the difference in patient volumes in the centers. Several authors have described a positive volume-outcome association for trauma patients [28–30]. In the current literature, there is no exact definition for high- or low-volume trauma centers, but most would consider UMCU a low-volume center, JHH a moderate, and HMC a high-volume center. In our opinion, in high-volume centers, three key factors attribute to better outcomes: the overall focus is better oriented toward the trauma patient, the presence of highly dedicated trauma teams, and the individual experience of the trauma surgeon. In the UMCU, 4 % of the admissions are trauma related, compared to 11 % at JHH and over 30 % at HMC [13, 14, 17]. Furthermore, the numbers of severely injured patients treated per trauma surgeon is substantially higher in JHH (>100 patients/surgeon) and HMC (>200 patients/surgeon) compared to UMCU (approximately 60 patients/surgeon).

Our data revealed a discrepancy in the causes of death demonstrated between the centers. The majority of patient deaths in all three centers were following CNS injuries, with JHH being more than 80 %. The cause is unclear, but may relate in part to transport times. There is a higher proportion of patients with neurotrauma in JHH (60.2 %), although the majority were only mild injuries (70 %). Whereas, the higher proportion of CNS injury deaths in UMCU (68 %) compared to HMC (60 %) could be partially explained by the higher proportion of patients with severe neurotrauma in the UMCU population (21 vs 18 %). The proportion of patients who died from exsanguination was the lowest at UMCU (5 %), which may be a consequence of the much smaller service area and shorter prehospital times.

Importantly, different cultural policies regarding withdrawal of treatment with inevitable death or brain death may exist at each institution, and might have distorted the in-hospital mortality rates. There were no essential differences in discharge destinations or supportive data that suggested different discharge rates to specialized high care facilities or hospices (data not shown here). In future research, long-term mortality, and nonfatal outcomes such as the quality of life among the survivors should be addressed to eliminate some of these potential biases.

Similarly, we observed a large difference in the number of ICU admissions between the trauma centers (Table 2). This can be explained by the different policies and the availability of ICU beds in the hospitals. While patients in various conditions are admitted at the ICU in HMC, a patient in UMCU or JHH is only admitted at the ICU in critical condition when close monitoring or ventilation is indicated. As presented in the results, the higher median ISS of the ICU patients in UMCU and JHH supports these thoughts.

Several other reasons could contribute to the differences in outcome and should be explored in future studies.

Our data have several weaknesses. There is a difference in the inclusion criteria for the trauma registries which could have influenced the results, but should be controlled by examine outcomes with ISS > 15 only. Elderly patients with an isolated neck of femur fracture are registered in the UMCU trauma registry, which may be a surrogate for unrecognized comorbidities [31]. Though, the number of these patients in UMCU is very low and therefore the influence on the analysis very minimal [32, 33].

This study demonstrated the demographic patterns and patient outcomes of trauma patients in Level I trauma centers in three international trauma systems. Besides the differences in patient characteristics and causes of death, a substantial difference in the mortality was demonstrated, mainly from neurological injury. Future research should reveal whether the outcome differences between the trauma centers still exist when nonfatal and long-term outcomes are compared. Furthermore, we must continue to benchmark and compare different trauma care systems with valid and reliable methods and identify strengths and weaknesses of systems in order to further inform trauma systems globally.
